# Impact of pelvic floor ultrasound in diagnosis of postpartum pelvic floor dysfunction: A protocol of systematic review

**DOI:** 10.1097/MD.0000000000021582

**Published:** 2020-08-07

**Authors:** Fan-bo Wang, Rong Rong, Jing-jun Xu, Guang Yang, Tian-you Xin, Xiao-hui Wang, Hai-bo Tang

**Affiliations:** a Department of Ultrasound, First Affiliated Hospital of Jiamusi University; b Department of Ultrasound, Wuxi No.2 People's Hospital, Wuxi; c Department of CT, Second Affiliated Hospital of Jiamusi University, Jiamusi, China.

**Keywords:** impact, pelvic floor dysfunction, pelvic floor ultrasound, postpartum

## Abstract

**Background:**

This study will appraise the impact of pelvic floor ultrasound (PFU) in diagnosis of postpartum pelvic floor dysfunction (PPPFD).

**Methods:**

Studies that report the impact of PFU in diagnosis of PPPFD will be examined in Cochrane Library, MEDLINE, EMBASE, PSYCINFO, Scopus, Web of Science, Allied and Complementary Medicine Database, CNKI, and WANGFANG up to June 1, 2020. Grey literature sources will also be searched. All potential case-controlled studies (CCSs) exploring the impact of PFU in diagnosis of PPPFD will be considered for inclusion in this study. Data will be extracted from eligible CCSs for data pooling and meta-analysis. Whenever necessary, we will also perform summary effect size, heterogeneity across studies, study quality assessment, and reporting bias.

**Results:**

The present study will estimate pooled outcome effects regarding the impact of PFU in diagnosis of PPPFD.

**Conclusion:**

This study may provide robust evidence to judge the impact of PFU on PPPFD

**Systematic review registration:**

PROSPERO CRD42020187623.

## 1 Introduction

Pelvic floor dysfunction (PFD) is a common disorder that affects ability to control and coordinate pelvic floor muscles.^[1–4]^ It constitutes a spectrum of pathologies^[5]^ and is associated with stress urinary incontinence (SUI), bowel incontinence, pelvic pain, sexual dysfunction, constipation, and pelvic organ prolapse (POP),^[6–9]^ which significantly affect quality of life for patients with PFD.^[10–12]^ It has been estimated that its prevalence varies from 23.7% to 46.2% of women experience at least 1 PFD,^[13,14]^ and its prevalence in females over 40 years old is between 30% and 50%.^[15]^ Its incidence is reported about 58.70% with about 48.3% for POP and 8.7% for SUI.^[16]^ PFD commonly affects women of all ages, but there is a higher risk for pregnancy women after delivery with PFD, also known as postpartum pelvic floor dysfunction (PPPFD).^[17–23]^ Thus, it is very important to diagnose this condition at early stage.

Pelvic floor ultrasound (PFU) is responsible for diagnosis of PPPFD, and a variety of studies have reported the impact of PFU for diagnosis of PPPFD.^[24–27]^ However, little is known about the impact of PFU in diagnosis of PPPFD at evidence-based medicine level. Thus, in order to better understand this issue, we will conduct a systematic review to address the impact of PFU in diagnosis of PPPFD.

## 2 Methods

### 2.1 Study registration

This study has been registered on PROSPERO with CRD42020187623. It has been reported following the guideline of Preferred Reporting Items for Systematic Reviews and Meta-Analysis Protocol statement.^[28]^

### 2.2 Inclusion criteria for study selection

#### 2.2.1 Type of studies

The present study will include case-controlled studies (CCSs) that assessed the impact of PFU in diagnosis of PPPFD.

#### 2.2.2 Type of participants

All adult female patients (over 18 years old) who were diagnosed as PPPFD will be included in this study, regardless educational background, economic status, and severity of PPPFD.

#### 2.2.3 Type of index test

Index test: PFU is used in detecting patients with PPPFD. However, we will exclude combination of PFU and other tests.

Reference test: patients who were detected by magnetic resonance imaging or computed tomography-proven PPPFD will be considered as comparators.

#### 2.2.4 Outcome measurements

Outcomes are sensitivity, specificity, positive likelihood ratio, negative likelihood ratio, and diagnostic odds ratio.

### 2.3 Data sources and search strategy

With the help of an academic librarian, this study will carry out a systematic literature search to find out studies that assess the impact of PFU in diagnosis of PPPFD. We will comprehensively search citations in Cochrane Library, MEDLINE, EMBASE, PSYCINFO, Scopus, Web of Science, Allied and Complementary Medicine Database, CNKI and WANGFANG up to June 1, 2020. In addition, grey literature sources, such as conference abstracts, thesis, and dissertation will be searched. All CCSs focusing on the impact of PFU in diagnosis of PPPFD will be included. We will provide search strategy of MEDLINE in Table 1. We will adapt similar search strategies to other electronic databases.

**Table 1:**
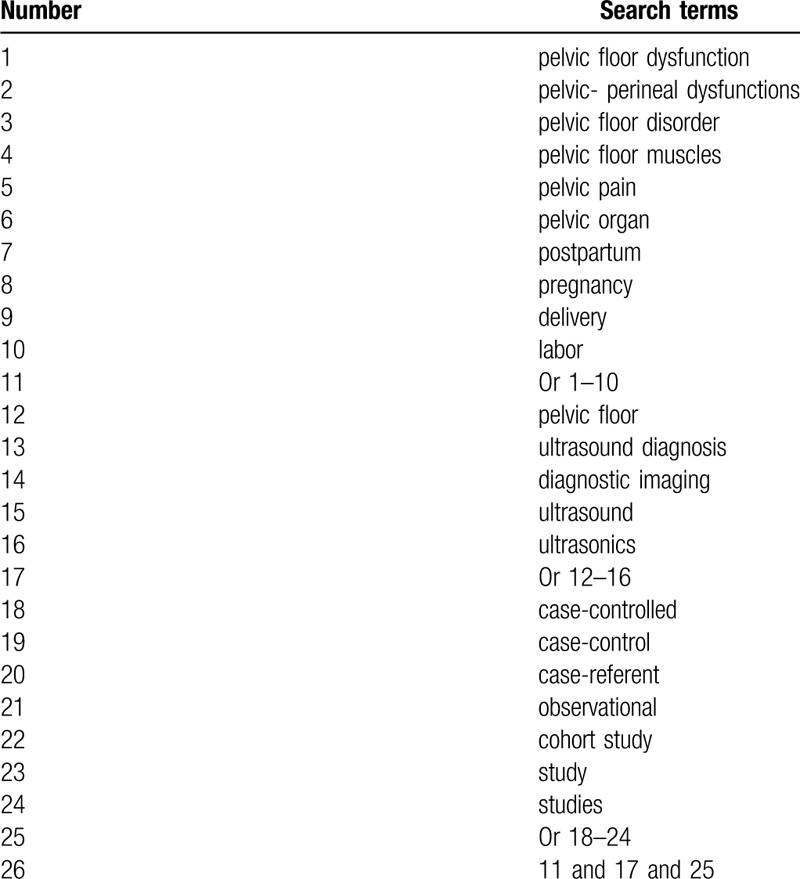
Search strategy applied in MEDLINE database.

### 2.4 Data collection and analysis

#### 2.4.1 Study selection

Two authors will scan titles and abstracts of studies retrieved utilizing the search strategy from electronic databases and grey literatures. All unconnected studies will be removed. Then, full text of potential studies will be retrieved for inclusion against all inclusion criteria. Any conflicts will be clarified through discussion with a third author. We will summarize study selection in a flow diagram.

#### 2.4.2 Data extraction

Two independent authors will extract data from all eligible studies utilizing data extraction sheet. It includes general information of included studies and patients (such as authors, title, time of publication, country, etc), sample size, inclusion and exclusion criteria, study quality, index and reference tests, and outcomes. Any disagreements will be resolved by a third author through discussion. If any missing or unclear information is identified, we will contact primary authors to request them.

### 2.5 Quality assessment

All eligible CCSs will be assessed by 2 independent authors using Quality Assessment of Diagnostic Accuracy Studies tool.^[29]^ Any opposition between 2 authors will be cleared up by a third author through discussion.

### 2.6 Statistical analysis

This study will apply RevMan V.5.3 software (London, UK) and Stata V.12.0 software (StataCorp; USA) to perform data analysis. We will summarize specific characteristics and study findings in tables. We will estimate outcome as descriptive statistics and 95% confidence intervals, and will perform plots of descriptive forest and summary receiver operating characteristic. Heterogeneity will be checked by *I*^2^ statistic. *I*^2^ ≤ 50% suggests low heterogeneity, and Mantel-Haenszel fixed-effects model will be used, while *I*^*2*^ > 50% indicates significant heterogeneity, and Mantel-Haenszel random-effects model will be applied. If there is low heterogeneity, we will conduct meta-analysis based on the sufficient eligible studies on the same outcome indicator. If there is substantial heterogeneity, we will carry out subgroup analysis to examine its possible sources.

### 2.7 Subgroup analysis

This study will perform subgroup analysis according to the different study characteristics, study qualities, and outcomes.

### 2.8 Sensitivity analysis

This study will conduct sensitivity analysis to examine stability of study findings by removing low quality studies.

### 2.9 Reporting bias

This study will test reporting bias using funnel plots and associated regression tests.^[30,31]^

### 2.10 Ethics and dissemination

This study will only extract data from published studies, thus no ethic approval is required. It will be published in a relevant peer-reviewed journal.

## 3 Discussion

Although many studies have reported the impact of PFU in diagnosis on PPPFD, no systematic review or/and meta-analysis is conducted to explore the impact of PFU in detection of PPPFD. Thus, this is the first systematic review to comprehensively search and summarize most recent evidence on the impact of PFU in diagnosis of PPPFD, and to synthesize the effect estimates from all included studies. The findings of this study will inform clinical practice and further studies focusing on the impact of PFU in diagnosis of PPPFD.

## Author contributions

**Conceptualization:** Tian-you Xin, Xiao-hui Wang.

**Data curation:** Fan-bo Wang, Rong Rong, Jing-jun Xu, Xiao-hui Wang.

**Formal analysis:** Rong Rong, Guang Yang, Tian-you Xin.

**Funding acquisition:** Xiao-hui Wang.

**Investigation:** Xiao-hui Wang.

**Methodology:** Fan-bo Wang, Rong Rong, Guang Yang, Tian-you Xin.

**Project administration:** Xiao-hui Wang.

**Resources:** Fan-bo Wang, Rong Rong, Jing-jun Xu, Guang Yang, Hai-bo Tang.

**Software:** Fan-bo Wang, Rong Rong, Jing-jun Xu, Guang Yang, Hai-bo Tang.

**Supervision:** Xiao-hui Wang.

**Validation:** Fan-bo Wang, Rong Rong, Guang Yang, Xiao-hui Wang, Hai-bo Tang.

**Visualization:** Fan-bo Wang, Jing-jun Xu, Tian-you Xin, Xiao-hui Wang.

**Writing – original draft:** Fan-bo Wang, Rong Rong, Guang Yang, Tian-you Xin, Xiao-hui Wang.

**Writing – review & editing:** Fan-bo Wang, Rong Rong, Jing-jun Xu, Xiao-hui Wang, Hai-bo Tang.

## References

[1] Dietz HP Female pelvic floor dysfunction--an imaging perspective. Nat Rev Gastroenterol Hepatol 2011;9:113–21. 22183184 10.1038/nrgastro.2011.213

[2] Mallett VT Bump RC The epidemiology of female pelvic floor dysfunction. Curr Opin Obstet Gynecol 1994;6:308–12. 7742490

[3] Bump RC Norton PA Epidemiology and natural history of pelvic floor dysfunction. Obstet Gynecol Clin North Am 1998;25:723–46. 9921553 10.1016/s0889-8545(05)70039-5

[4] Sung VW Hampton BS Epidemiology of pelvic floor dysfunction. Obstet Gynecol Clin North Am 2009;36:421–43. 19932408 10.1016/j.ogc.2009.08.002

[5] Turner CE Young JM Solomon MJ Incidence and etiology of pelvic floor dysfunction and mode of delivery: an overview. Dis Colon Rectum 2009;52:1186–95. 19581867 10.1007/DCR.0b013e31819f283f

[6] Gao L Ding S Ding Y Symptom distribution of female pelvic floor dysfunction patients with constipation as chief complaint. Zhonghua Wei Chang Wai Ke Za Zhi 2018;21:798–802. 30051449

[7] Ramage L Georgiou P Qiu S Can we correlate pelvic floor dysfunction severity on MR defecography with patient-reported symptom severity? Updates Surg 2018;70:467–76. 29255962 10.1007/s13304-017-0506-0 PMC6244712

[8] Haylen BT Maher CF Barber MD An International Urogynecological Association (IUGA)/International Continence Society (ICS) joint report on the terminology for female pelvic organ prolapse (POP). Neurourol Urodyn 2016;35:137–68. 26749391 10.1002/nau.22922

[9] DeLancey JO The anatomy of the pelvic floor. Curr Opin Obstet Gynecol 1994;6:313–6. 7742491

[10] Zhu Q Shu H Dai Z Effect of pelvic floor dysfunction on sexual function and quality of life in Chinese women of different ages: an observational study. Geriatr Gerontol Int 2019;19:299–304. 30811813 10.1111/ggi.13618

[11] Ascanelli S Morganti L Martinello R Combined rectal and gynecologic surgery in complex pelvic floor dysfunction: clinical outcomes and quality of life of patients treated by a multidisciplinary group. Minerva Chir 2018;73:345–7. 29911796 10.23736/S0026-4733.18.07429-1

[12] Frota IPR Rocha ABO Neto JAV Pelvic floor muscle function and quality of life in postmenopausal women with and without pelvic floor dysfunction. Acta Obstet Gynecol Scand 2018;97:552–9. 29352460 10.1111/aogs.13305

[13] MacLennan AH Taylor AW Wilson DH The prevalence of pelvic floor disorders and their relationship to gender, age, parity and mode of delivery. Br J Obstet Gynaecol 2000;107:1460–70. 10.1111/j.1471-0528.2000.tb11669.x 11192101

[14] Nygaard I Barber MD Burgio KL Prevalence of symptomatic pelvic floor disorders in US women. JAMA 2008;300:1311–6. 18799443 10.1001/jama.300.11.1311 PMC2918416

[15] Lawrence JM Lukacz ES Nager CW Prevalence and co-occurrence of pelvic floor disorders in community-dwelling women. Obstet Gynecol 2008;111:678–85. 18310371 10.1097/AOG.0b013e3181660c1b

[16] Su Y Li D Han Y The screening of pelvic floor disordersamong women attending health examination in Zhongshan city. Guangdong Med J 2014;35:1405–7.

[17] Chen GD Pelvic floor dysfunction in aging women. Taiwan J Obstet Gynecol 2007;46:374–8. 18182342 10.1016/S1028-4559(08)60006-6

[18] Harvey MA Pelvic floor exercises during and after pregnancy: a systematic review of their role in preventing pelvic floor dysfunction. J Obstet Gynaecol Can 2003;25:487–98. 12806450 10.1016/s1701-2163(16)30310-3

[19] Rørtveit G Hannestad YS Association between mode of delivery and pelvic floor dysfunction. Tidsskr Nor Laegeforen 2014;134:1848–52. 25314985 10.4045/tidsskr.13.0860

[20] Zuchelo LTS Bezerra IMP Da Silva ATM Questionnaires to evaluate pelvic floor dysfunction in the postpartum period: a systematic review. Int J Womens Health 2018;10:409–24. 30123009 10.2147/IJWH.S164266 PMC6087030

[21] Chen Z Huang H Chen QY Effect of modified Buzhong Yiqi decoction combined with pelvic floor muscle exercise-biofeedback-electrical stimulation on early stage postpartum pelvic floor dysfunction. Zhongguo Zhong Yao Za Zhi 2018;43:2391–5. 29945396 10.19540/j.cnki.cjcmm.20180305.001

[22] Sun Z Zhu L Lang J Postpartum pelvic floor rehabilitation on prevention of female pelvic floor dysfunction: a multicenter prospective randomized controlled study. Zhonghua Fu Chan Ke Za Zhi 2015;50:420–7. 26311549

[23] Pregazzi R Sartore A Bortoli P Immediate postpartum perineal examination as a predictor of puerperal pelvic floor dysfunction. Obstet Gynecol 2002;99:581–4. 12039115 10.1016/s0029-7844(01)01763-x

[24] Leombroni M Buca D Liberati M Post-partum pelvic floor dysfunction assessed on 3D rotational ultrasound: a prospective study on women with first- and second-degree perineal tears and episiotomy. J Matern Fetal Neonatal Med 2019;1–1. ahead of print. 10.1080/14767058.2019.1609932 31291792

[25] Laterza RM Schrutka L Umek W Pelvic floor dysfunction after levator trauma 1-year postpartum: a prospective case-control study. Int Urogynecol J 2015;26:41–7. 25007898 10.1007/s00192-014-2456-0

[26] van Delft K Sultan AH Thakar R The relationship between postpartum levator ani muscle avulsion and signs and symptoms of pelvic floor dysfunction. BJOG 2014;121:1164–71. 24548759 10.1111/1471-0528.12666

[27] Rogers RG Leeman LM Borders N Contribution of the second stage of labour to pelvic floor dysfunction: a prospective cohort comparison of nulliparous women. BJOG 2014;121:1145–53. 24548705 10.1111/1471-0528.12571 PMC4565727

[28] Shamseer L Moher D Clarke M PRISMA-P Group. Preferred reporting items for systematic review and meta-analysis protocols (PRISMA-P): elaboration and explanation. BMJ 2015;349:g7647. 10.1136/bmj.g7647 25555855

[29] Whiting PF Rutjes AW Westwood ME QUADAS-2: a revised tool for the quality assessment of diagnostic accuracy studies. Ann Intern Med 2011;155:529–36. 22007046 10.7326/0003-4819-155-8-201110180-00009

[30] Sutton AJ Duval SJ Tweedie RL Empirical assessment of effect of publication bias on meta-analyses. BMJ 2000;320:1574–7. 10845965 10.1136/bmj.320.7249.1574 PMC27401

[31] Egger M Davey Smith G Schneider M Bias in meta-analysis detected by a simple, graphical test. BMJ 1997;315:629–34. 9310563 10.1136/bmj.315.7109.629 PMC2127453

